# Nanocrystalline silicon thin film growth and application for silicon heterojunction solar cells: a short review

**DOI:** 10.1039/d0na00791a

**Published:** 2021-05-17

**Authors:** Mansi Sharma, Jagannath Panigrahi, Vamsi K. Komarala

**Affiliations:** Centre for Energy Studies, Indian Institute of Technology Delhi New Delhi-110016 India mansisharma663@gmail.com

## Abstract

Doped nanocrystalline silicon (nc-Si:H) thin films offer improved carrier transport characteristics and reduced parasitic absorption compared to amorphous silicon (a-Si:H) films for silicon heterojunction (SHJ) solar cell application. In this article, we review the growth conditions of nc-Si:H thin films as the carrier-selective layers for SHJ solar cells. Surface and growth zone models are analysed at different stages of incubation, nucleation, and growth of the silicon nanocrystallites within the hydrogenated amorphous silicon matrix. The recent developments in the implementation of nc-Si:H films and oxygen-alloyed nc-SiO_*x*_:H films for SHJ cells are highlighted. Furthermore, hydrogen and carbon dioxide plasma treatments are emphasised as the critical process modification steps for augmenting the nc-Si:H films' optoelectronic properties to enhance the SHJ device performance with better carrier-selective interfaces.

## Abstract 2

Doped nanocrystalline silicon thin films, in which silicon nanocrystallites are embedded in an amorphous silicon matrix, are emerging as carrier-selective contacts for next-generation silicon heterojunction solar cells.

## Graphical abstract

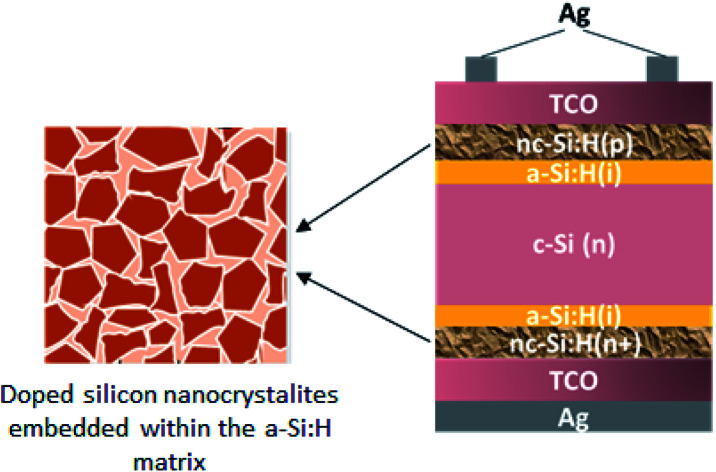

## 1. Introduction

Solar photovoltaics (SPV) is one of the best options to meet the world's terawatt power demand in the near future.^1^ Silicon-wafer based solar cells with high power conversion efficiency (PCE) and cost reduction have been driving the technological advancements in this area. These are based on the classical homo-junction solar cells processed at high temperature, and the amorphous/crystalline silicon (a-Si/c-Si) heterojunction (SHJ) solar cells prepared at low temperature with relatively better performance. In the silicon family, the hydrogenated amorphous silicon (a-Si:H) has drawn attention with its atypical optoelectronic properties (tailored forbidden energy bandgap, carrier mobility, and conductivity).^2^ The concept of a-Si:H/c-Si heterojunction (SHJ) has emerged based on the c-Si and the a-Si:H materials' comprehensive understanding, the power conversion efficiency (PCE) of such SHJ designs has surpassed 25% for both-side contacted cells,^3^ and the interdigitated back contact (IBC) design has led to >26% efficient cells.^4^ The PCE of a both-side contacted SHJ solar cell is limited mainly by the short-circuit current (*J*_SC_) losses in the amorphous layers.^5–7^ A novel device architecture, *e.g.*, rear-emitter device configuration aided with new functional materials, is now considered essential for the SHJ device performance augmentation.

The *J*_SC_ of a both-side contacted SHJ cell has been recognised to be limited by the optical losses such as (1) parasitic absorption in the short wavelength region (<500 nm) by the a-Si:H layers on the front side, thereby reducing the *J*_SC_ by ∼1.5 mA cm^−2^,^5,6,8^ and (2) refractive index (*n*, at 632 nm unless specified otherwise) mismatch among c-Si (*n* ∼ 3.8), a-Si (*n* ∼ 4.0), and the transparent conducting oxide (TCO) (*n* ∼ 2.0) at the front which may result in some optical reflection losses.^8^ Moreover, the highly defective a-Si:H(p) layer has slightly inferior electronic properties,^9^*e.g.* dark conductivity (*σ*_0_) ∼ 10^−2^ S cm^−1^, because of a low doping efficiency leading to a high series resistance as well as inadequate a-Si(p)/TCO electronic contact, and hence a low fill factor (FF) of the SHJ device.^10^ Hydrogenated amorphous silicon oxide (a-SiO_*x*_:H) has been implemented as an alternative material for SHJ cells to minimise the front region optical losses due to its improved transparency.^11,12^

Recently, research efforts have been shifted toward doped nanocrystalline silicon (nc-Si:H) thin films as carrier-selective layers for SHJ cells.^13–16^ In nc-Si:H film, silicon nanocrystallites are embedded in a matrix of amorphous silicon, and H is preferentially located at the grain boundaries or in the amorphous phase.^17,18^ With added crystallinity, an increase in transparency as well as conductivity of the silicon film is expected. The optoelectronic properties of nc-Si:H strongly depend on the crystalline fraction. Because of the mixed crystalline phase, the nc-Si:H film has higher doping efficiency, so *σ*_0_ can be higher by more than two orders of magnitude than that of the doped a-Si:H.^15,17,19^ Combined with improved carrier-transport properties, it could help improve the FF of the SHJ cell.^15^ The nc-Si:H thin film can reduce parasitic absorption at the shorter wavelength region due to its higher optical band gap (*E*_04_ ∼ 2.0 eV) compared to the a-Si:H films, which would enable an increase of the photocurrent of the SHJ cell. Additionally, nc-Si:H has been reported to suppress the Schottky-barrier effect which is generally observed between a-Si:H(p) and the adjacent TCO layer.^15^ The refractive index of the nc-Si:H film (*n* ∼ 3.4) is lower than that of a-Si:H and depends on the crystalline fraction and doping concentration.^17–19^ Moreover, with the CO_2_ plasma during the nc-Si deposition,^2^ a lower refractive index (*n* ∼ 2.8) can be achieved from the oxygen-alloyed nc-Si:H (or, nc-SiO_*x*_:H) layer, which can significantly improve the light coupling at the front side of the SHJ cell,^20–23^ yielding *J*_SC_ above 40 mA cm^−2^ for the first time for a two-side contacted SHJ cell.^23^ Recently, large-area (244 cm^2^) SHJ solar cells featuring nc-SiO_*x*_:H(n) front contacts have been demonstrated with a power conversion efficiency of 23.1% (ref. 24) and a certified 25.11% efficient cell by Hanergy.^25^

Achieving the aforementioned improved properties of the nc-Si:H layers during application in the SHJ solar cell, where the deposited layers' thickness is usually maintained below 10 nm, is a challenging job. Several requirements need to be addressed: (1) fast nucleation and suppression of the “incubation zone” amorphous layer during deposition, (2) sufficiently high crystalline fraction for a high doping efficiency and electronic conductivity, (3) preserving a high passivation level/interface from the underlying very thin (∼5 nm) intrinsic a-Si:H layer (this is a stringent requirement during the nc-Si growth, which enables high open-circuit voltage and efficiency of the SHJ cell). Moreover, for industrial manufacturing, a thin layer with a short deposition time (<100 s) is necessary to keep the production costs low. Hence, the deposition rate has to be greater, or a very thin (∼10 nm) layer has to satisfy the material functionalities. Furthermore, the boron dopant has an amorphising impact on the layer growth, inhibiting the fast nucleation of the p-type nc-Si:H layer.^26^ Therefore, deposition of a high-quality thin nc-Si:H layer with these stringent requirements is a challenging task. SHJ devices, when fabricated in the inverted-polarity configuration, wherein the p-type emitter is located on the rear side while the illumination side features the n-type a-Si:H or nc-Si:H or nc-SiO_*x*_ electron-contact layers, relax some of the constraints and also lead to better cell results.^10,14,15,24,25^ In this article, we review the growth conditions necessary to realise very thin high-quality nc-Si films, their optoelectronic properties, and the integration of the films as carrier-selective contacts in the SHJ solar cells. Furthermore, we present information related to the possibilities of process modifications based on the deposition techniques and plasma treatments for tailoring the nc-Si layers' optoelectronic properties.

## 2. Nano-/micro-crystalline silicon thin films

Hydrogenated nano-/micro-crystalline silicon (nc-/μc-Si:H) is a biphasic material, with the nature of the film being categorized based on the nanocrystallites' distribution/size embedded in the amorphous silicon matrix.^17,18^ The crystallite size variation during the growth process has been understood based on the classical theory of crystal growth and nucleation, which depicts a direct relation between the critical crystal size and atomic volume.^27^ If the occupancy of crystallites is dense and the size is a few nanometers, the film is considered nanocrystalline. Films with larger silicon crystallites (>10 nm) are considered as micro-crystalline.^18^

**Fig. 1:**
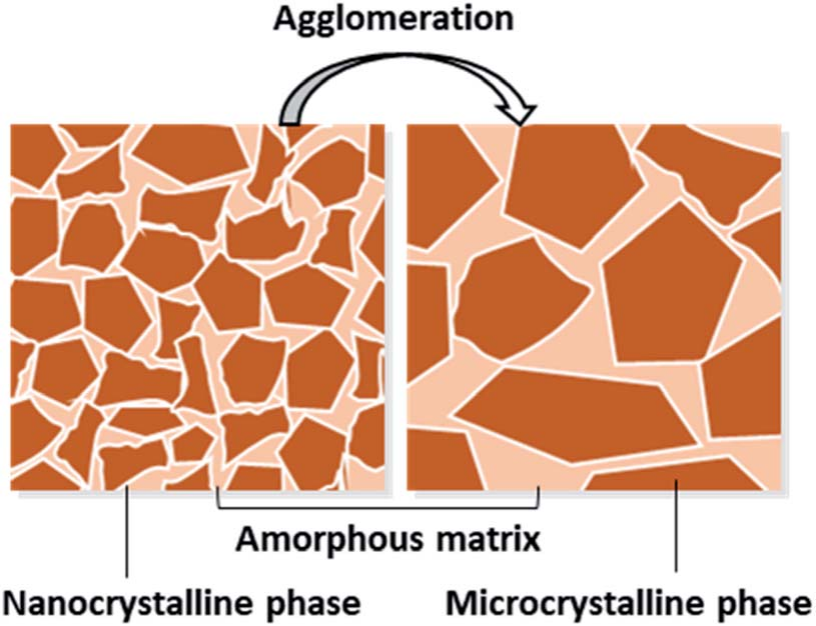
Schematic representation of nano- and micro-crystalline silicon within the amorphous silicon matrix.

The schematic in Fig. 1 depicts the segregation of nano- and micro-crystalline phases within the amorphous matrix. Depending on the parametric conditions, the agglomeration of fine silicon nanocrystallites leads to the formation of microcrystallites. To effectively probe the presence of such crystallites in a film, Raman spectroscopy (structural) and electron microscopy (morphology) characterisation tools are adopted.^28^ However, the silicon crystallinity variation results in the formation of complex phases within the amorphous matrix. Investigating and mapping the structural details of such complex phases with optoelectronic properties of films is indeed a tedious task. For crystallite distribution analysis, other factors such as effective crystalline volume fraction, structural parameters, and dielectric functions need to be considered.^29^

### 2.1 Growth conditions for crystallization

**Fig. 2:**
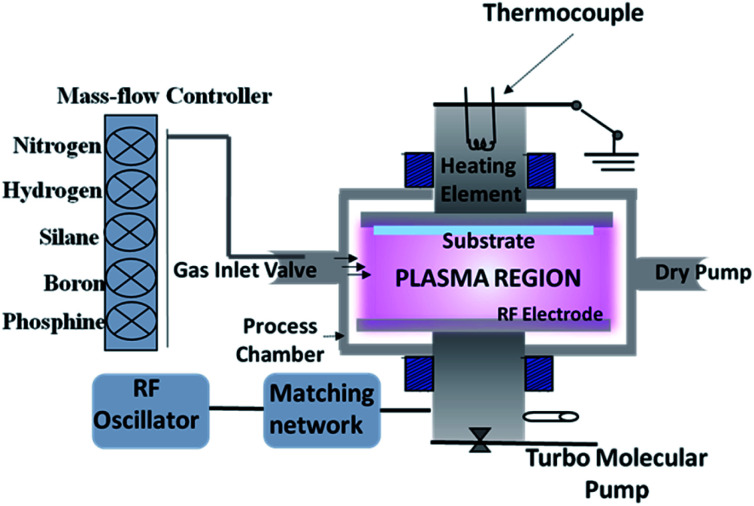
Schematic of a basic plasma enhanced chemical vapor deposition system.

**Fig. 3:**
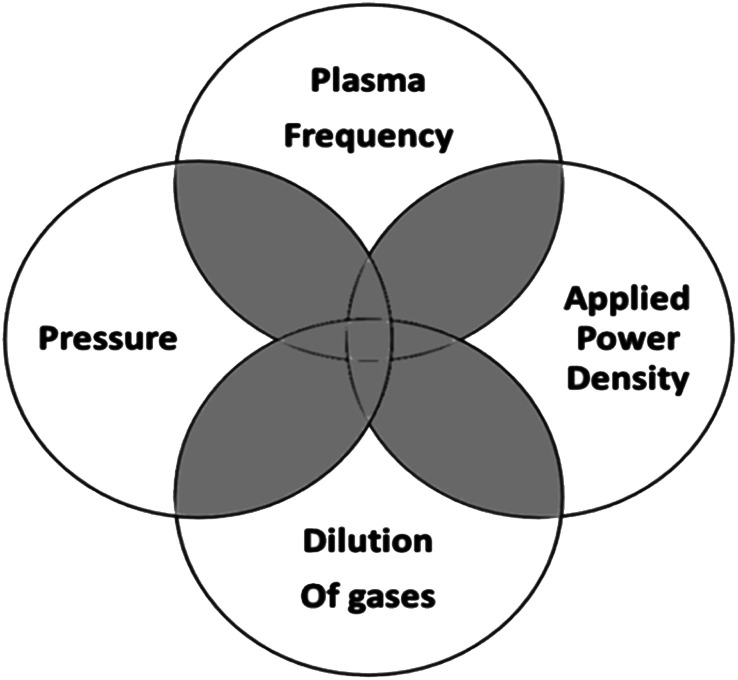
Various deposition conditions for the growth of nanocrystalline silicon thin films.

For deposition of amorphous and microcrystalline Si:H films, the plasma-enhanced chemical vapor deposition (PECVD) system in a simple parallel-plate configuration is commonly used.^2,17,18^ In a PECVD process, various primary reactions and some secondary reactions take place.^30^ The a-Si:H film growth usually occurs at a normal pressure of <1 torr, with an RF plasma excitation frequency of 13.56 MHz (or VHF), in a discharge of silane and other dopant gasses. The schematic of a basic PECVD system is shown in Fig. 2. It consists of a vacuum system, precursor gas handing system, RF power supply, impedance matching network, and a set of parallel electrodes – one being the RF electrode and the other the substrate holder heated to the desired temperature (<300 °C). This technique's basic principle follows from the dissociation of the precursor gas molecules by the energetic electrons present in the plasma, generation of mainly silyl (SiH_3_) radicals and ions, diffusion of these radicals towards the substrate, and bonding with surface dangling bonds resulting in film growth. The nc-Si:H film deposition involves similar processing steps to those required for the a-Si:H film, but the deposition conditions are different. To induce crystallisation in an amorphous silicon matrix, some general deposition conditions need to be fine-tuned, which are very interdependent, as depicted in Fig. 3. The crystallisation can be initiated either by variation of one of the parameters or a combination of parameters represented by the shaded region in Fig. 3. For instance, along with a flux of SiH_3_ radicals, a large flux of atomic hydrogen toward the substrate is required to induce crystallinity.^17,18,30^ Therefore, silane is highly diluted with hydrogen for deposition of nc-Si:H. Another requirement is deposition temperature; usually, higher temperature favours crystallinity.

The independent parameter optimisation (excitation frequency, power density, gas pressure, and hydrogen dilution) is considered a favourable condition for the nc-Si:H films' growth.^31–34^ Guo *et al.*^31^ have explored the evolution of μc-Si:H thin films with the variation of deposition pressure. The study has highlighted the suitable role of working pressure and SiH_4_ depletion combination for attaining a film's high deposition rate. Veneri *et al.*^32^ have investigated the role of high-frequency plasma along with power density variation in μc-Si:H film deposition, and a transition from the a-Si:H to the μc-Si:H phase was observed with the variation of power. Rath *et al.*^33^ have investigated the effect of gas pressure variation on the electron density and energy of plasma, which has revealed the role of plasma species in modifying the microstructure of a film. The cyclic or layer-by-layer (LBL) deposition method, in which the substrate is alternately exposed to silane and hydrogen plasmas, has been explored for the deposition of μc-Si:H films.^34^ With this process, the transition from an amorphous to a crystalline phase could be achieved by suitable hydrogen and silane plasma exposure times. With increasing the number of cycles, the growth zone gradually changes from incubation to nucleation and growth, with an increasing crystalline fraction, and once the steady-state phase is reached, the entire film down to the interface becomes micro-crystalline. The efficacy of the LBL process is that it entirely avoids the silyl ion bombardment during hydrogen plasma exposure, therefore promoting hydrogen-induced crystallisation.

The growth of nc-Si involves four phases: incubation, nucleation, growth and steady-state, represented by increasing crystalline fraction.^34^ The amorphous incubation phase during a film growth is the initial stage, followed by the onset of nucleation of crystallites.^34–36^ This incubation phase is considered a critical factor for controlling the film's crystalline fraction.^18,33^ The thickness of this incubation zone has to be governed by the deposition parameters. The underlying intrinsic a-Si:H layer is prone to induce a thicker incubation zone. Specific plasma conditions are known to reduce the incubation zone's thickness, but can also cause the passivation of the a-Si/c-Si interface to deteriorate.

**Fig. 4:**
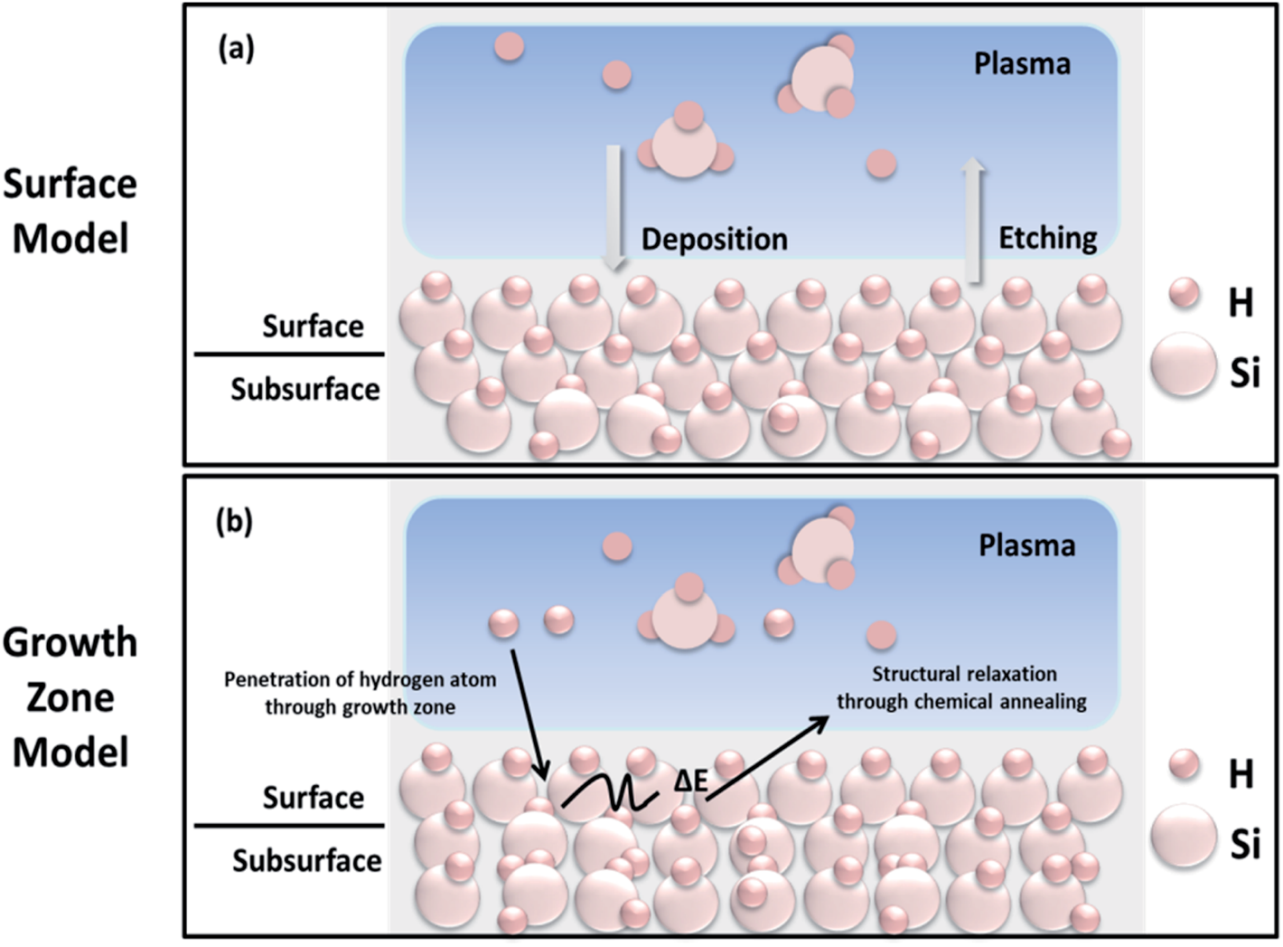
Schematic representation of (a) the surface model and (b) growth zone models for the growth of nc-Si:H thin films.^18,30^

A few growth models have been considered for explaining the origin of crystallinity.^35–40^ Based on similarities among these models, they are put into two groups, which are (1) surface model, and (2) growth-zone model.^18,38,39^Fig. 4 shows the schematic representation of the two models. The surface model explains the films' growth dependence on plasma species interaction with the material's surface, since species present in the plasma discharge will have quite different energies.^42^ In this model, the adsorbed radicals can diffuse across the surface until they find the energetically favourable site, leading to the formation of crystallites *via* structural rearrangement, and the model therefore depends on the surface mobility of the precursors. Additionally, the surface model considers the selective etching of silicon by hydrogen ions, and the etch rate is higher for the disordered phase in comparison with the ordered phase.^35,40,41^ The process of successive deposition and selective etching leaves the surface and bulk with distributed voids, and hence the structure is considered to remain porous. The etching of the disordered surface can enhance the proportion of ordered geometry with better crystallisation. The simultaneous deposition and etching processes also lead to the formation of unstable nuclei during crystallisation. Consequently, the surface model approach restricts the discussion related to the details of subsurface growth and nucleation processes during film deposition, and is thus suggested to give a limited explanation of a film's growth.

Conversely, in the growth-zone model, the formation of crystalline phases results from the interaction of atomic hydrogen ions with an a-Si layer beneath the surface (subsurface);^42–44^ the extension of the growth zone is only a few nanometers below the surface. Thus, the dependency of incubation time and the crystalline fraction is highly associated with hydrogen concentration. The hydrogen ions react with the film's subsurface strained/weak Si–Si and Si–H bonds and promote structural relaxation and rearrangement of the silicon network (crystal nucleation) by the generation of heat during chemical reactions (called chemical annealing).^43^

Apart from diluting the silane plasma with hydrogen during the film deposition, the post-deposition moderate hydrogen plasma treatment (HPT) is also considered one of the crucial process steps to promote crystallinity *via* surface modification.^45^ HPT promotes the structural rearrangement of the disordered state into an ordered state by H insertion *via* the process of chemical annealing.^35^ The motivation behind the plasma treatment has been to add an extra degree of freedom to the growth zone by separating the growth and etching processes. With HPT, the effective diffusion of atomic hydrogen (in the absence of silane plasma) promotes the incubation phase effectively at the initial stage of film growth. The formation of a porous silicon network in the initial stage of film growth has also been suggested as the necessary condition for the nucleation process. Therefore, the nucleation of crystallites is considered to be dependent on the composition of the initial seed layer. The diffusion of hydrogen during the growth of intrinsic/doped nc-Si:H thin films needs analysis with the surface/growth zone model's help to understand the interfaces and surface bonding. The incubation phase also depends on the substrate selectivity/orientation apart from the plasma gas composition and hydrogen plasma treatment as emphasised in the work of ref. 39, where the study also highlights the role of alternate HPT and amorphous silicon growth by the LBL deposition technique, thereby segregating the deposition and etching conditions.

Similar to HPT, the discontinuous plasma treatment has also been implemented to explore film growth near the transition zone (between incubation and nucleation).^46,47^ In a recent study, we have demonstrated the structural modifications of the a-Si:H/nc-Si:H film using a pulsed plasma during the CVD process.^48^ The importance of pulsed plasma has been verified in terms of an extended transition zone, and the growth of a typical sub-nanocrystalline phase has been explored by Raman spectroscopy. The study showed a correlation between raising stress and Raman peak broadening as a consequence of structural modification with an improvement in crystallinity. The optical and electrical characteristics are also well correlated with the structural properties of the nc-Si:H film,^49^ like the combination of a large bandgap and high electrical conductivity in comparison to the pure a-Si:H film. So, pulsed plasma processing can also be an effective way to explore the overlooked growth zone during film deposition.

## 3. Progress in the SHJ cell fabrication using nc-Si:H layers as the carrier-selective contacts

**Fig. 5:**
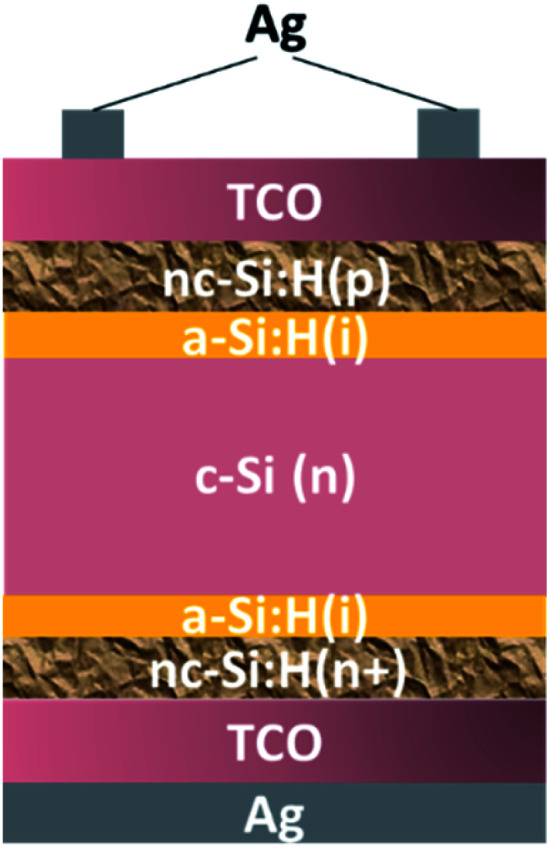
Schematic of a SHJ solar cell featuring doped nc-Si:H layers as carrier-selective contacts.

For SHJ solar cell fabrication, the initial growth and the related phases of the carrier-selective layers are of great importance; the schematic of a SHJ cell based on the nc-Si:H carrier-selective layers is presented in Fig. 5. In comparison to the homojunction silicon solar cells, an unoptimized SHJ cell shows an inferior performance due to the a-Si:H/c-Si hetero-interface, which will introduce energy band offsets and interface/surface defect states leading to anomalous behaviour in the light current density–voltage (*J*–*V*) characteristics and the carrier transport recombination, respectively.^50^ So, apart from understanding the growth process of the nc-Si:H thin films (for an SHJ solar cell application), there is a need to develop a better nc-Si/c-Si interface for facilitating charge carrier collection. Another critical consideration in an SHJ cell fabrication is c-Si surface passivation by a very thin (∼5 nm) intrinsic a-Si:H layer. For the purpose of better surface passivation, one needs to avoid an epitaxial passivation layer growth on the c-Si surface for minimizing the interface/surface defect states (which promotes the carrier recombination).^51,52^

Apart from avoiding an epitaxial growth at the a-Si:H(i)/c-Si interface, efficient doping in the carrier-selective layers is also considered a critical factor in augmenting the device performance.^53^ The effective doping of a carrier-selective layer in a device facilitates (1) band bending, (2) better field-effect passivation, and also (3) reduces series resistance as well as contact resistivity with the adjacent TCO layer.^54^ The significant advantage of the nc-Si:H film is its high doping efficiency in comparison with the a-Si:H film,^17,55^ which also facilitates suppression of excess defect formation in a film.^56^ In the following sections, we highlight the research work on nc-Si:H thin films for SHJ device application as the carrier-selective layers.

### 3.1 Optical properties

For enhancing the photogeneration of charge carriers in the c-Si absorber, one has to suppress the optical absorption in the front carrier-selective layers. The localised bulk/surface defect states (from the mixture of strained, broken and dangling bonds) of a thin a-Si:H/nc-Si:H film near the middle of the energy bandgap influence the optical absorption.^56^ Since the nc-Si:H film consists of both the amorphous and the nanocrystalline phases, the optical absorption of such biphasic film is lower for high energy photons in comparison with the a-Si:H film due to the optical bandgap of >1.9 eV,^57–59^ from the quantum size effects.^60^ For enhancing the optical transparency further, the nc-SiO_*x*_:H thin films have also been utilised, where the inclusion of oxygen reduces the optical absorption without affecting the charge carrier transport.^2,20–25,60,61^

Mazzarella *et al.*^23^ have demonstrated SHJ solar cells incorporating the p-type μc-SiO_*x*_:H emitter layer with a power conversion efficiency of 20.3% and *J*_SC_ exceeding 40 mA cm^−2^. The work highlighted the advantage of the μc-SiO_*x*_:H emitter layer over the conventional a-Si:H layer because of the improved refractive index matching of the front layer stack (better light coupling) and parasitic absorption reduction leading to a gain in *J*_SC_ of ∼1.7 mA cm^−2^. However, the study remained open-ended with scope for improving the cell's series resistance and fill factor depending on the structural changes and size of crystallites, which eventually helps in the charge carrier transport. Later, in a similar investigation, n-type nc-SiO_*x*_:H layers with a range of refractive indices and conductivities were implemented as the front carrier-selective contacts in a rear-emitter configuration to avoid the optical constraints and to reduce the front contact resistance with the TCO.^61,62^ The study demonstrated the advantage of the nc-SiO_*x*_:H(n) layers as the front contact and the rear-emitter configuration of the SHJ solar cell leading to a best conversion efficiency of 21.6% (*J*_SC_ = 40.0 mA cm^−2^, *V*_OC_ = 729 mV, FF = 80.0%). Recently, the advantage of a 20 nm thick nc-SiO_*x*_:H(n) layer as a front contact layer has been demonstrated in a certified record 25.11% efficient large-area (244 cm^2^) SHJ solar cell of Hanergy, while also utilising other improved functionalities such as bilayer intrinsic a-Si passivation, fine printing, environment control, *etc.*^25^ The benefit of the front nc-SiO_*x*_:H(n) contact layer is seen from the 39.55 mA cm^−2^*J*_SC_ value and a very high FF (∼85%). This study shows that it is possible to implement thicker front contacts in SHJ solar cells with better crystallinity as well as transparency.

### 3.2 Carrier transport and crystallinity

**Fig. 6:**
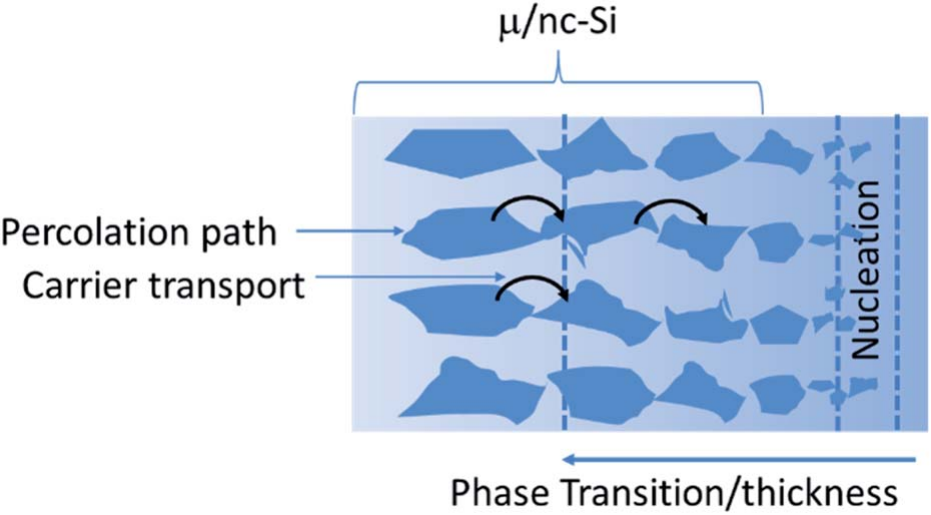
Schematic representation of film thickness dependent carrier transport mechanism *via* silicon micro-/nano-crystallites embedded in an amorphous silicon matrix.^23^

The shape and size of the silicon nanocrystallites as well as the crystalline fraction, are considered to play a significant role in allowing a conductive percolation path for the charge carriers.^23,63^ A critical threshold for percolation has been identified at a crystalline fraction of 0.2–0.3, and a sublinear dependence of the Hall mobility on the crystallite size has also been determined for the nanocrystalline films.^63^ Because of the directional evolution, a columnar or filament structure of the crystallites is suggested to be beneficial for a low threshold of percolation.^64^ As the growth of the nc-films is abruptly terminated, and the crystallite size varies with thickness, the shape of the crystallites is also suggested to be conical for such ultrathin films.^15^ The thickness of the filaments/columns could be estimated to be about 5 nm from TEM or atom probe tomography (APT) measurements.^15,62,64^ Therefore, the effective carrier transport mechanism is determined by the nanocrystal's randomness/distribution, size and density. The typical dependence of the crystalline fraction on the film thickness is presented in Fig. 6, which depicts an increase in crystallite size and fraction in the growth direction with an increase in film thickness.

**Fig. 7:**
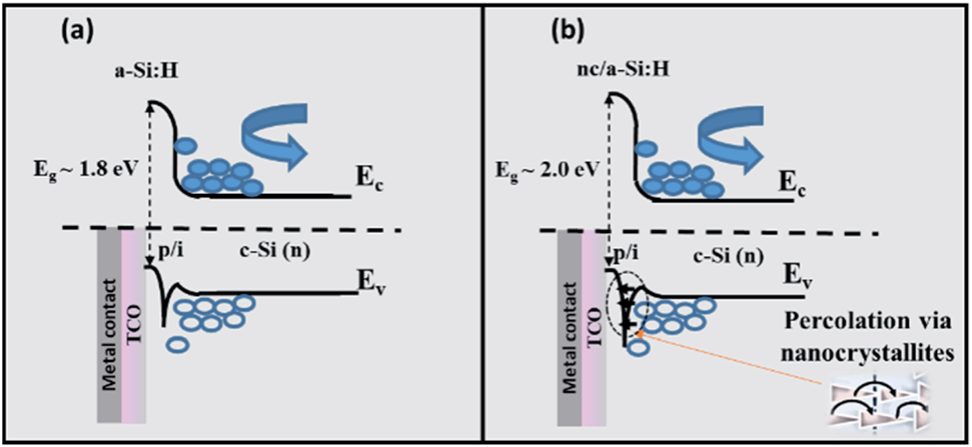
Energy band diagrams of (a) a-Si:H(p)/a-Si:H(i)/c-Si and (b) nc-Si:H(p)/a-Si:H(i)/c-Si heterostructures representing the charge carrier transport variation.

Doped nc-Si:H layers are considered to minimise the associated transport restrictions in an SHJ device.^55^ This is generally related to a better band bending of c-Si at the interface because of the enhanced crystallinity as well as doping.^65^ However, efficient p-type doping is challenging to realise in nc-Si films as it causes increased optical absorption and suppression of nucleation.^66^ The crystallinity as well as p-type doping efficiency may depend on the type of dopant, *e.g.*, trimethyl boron (TMB), boron trifluoride (BF_3_) or diborane (B_2_H_6_).^66^ For example, TMB reportedly causes delayed nucleation because of the generation of methyl radicals during deposition or the atomic H neutralising the B dopants. B_2_H_6_ and BF_3_ have been previously shown to cause better inclusion of active dopants. Fig. 7 illustrates the junction formation and energy band alignment of the SHJ cells with the a-Si:H(p)/a-Si:H(i)/c-Si and nc-Si:H(p)/a-Si:H(i)/c-Si heterostructures. The widening of nc-Si's energy bandgap can influence the conduction and valence band offsets at the interface, and the nc-Si helps in minority carrier transport by providing a percolation path *via* the nanocrystallites within the a-Si matrix.

Nogay *et al.*^15^ reported efficient incorporation of doped nc-/μc-Si:H layers into one of the sides as the carrier-selective contacts in the SHJ cells demonstrated improved carrier transport and contact resistivity. The experiments revealed substantial lowering of the contact resistivity of doped nc-Si layers, and a significant reduction of activation energies (from 0.284 to 0.077 eV) in comparison with the a-Si:H layers, which suggests suppression of the Schottky-barrier effect. Replacing the doped a-Si:H layers with the nc-Si:H counterparts enabled up to 1.5% gain in FF of the SHJ solar cell. Good power conversion efficiencies of 20.9% and 21.1% were achieved by utilising front side n-type nc-Si:H and rear side p-type nc-Si:H layers from an SHJ device, respectively, while maintaining V_OC_ values of 720 mV. Kirner *et al.*^67^ have demonstrated the implementation of a front nc-Si:H(p)/nc-SiO_*x*_:H(p) contact stack and addressed the importance of the additional very thin (3 nm) nc-Si:H(p) contact layer over the emitter toward the front TCO for maximising the FF (minimising the series resistance) by avoiding the dominant loss mechanism at the front TCO/emitter interface and highlighted the role of nc-Si:H layers' low activation energies. Richter *et al.*^64^ have explored nc-SiO_*x*_:H layers as the carrier-selective layers in a SHJ solar cell by varying the optical bandgap from 1.9 to 2.9 eV and refractive index from 1.9 to 3.6 in the simulations. Table 1 shows some of the SHJ device photovoltaic parameters featuring the nc-Si:H and nc-SiO_*x*_:H carrier selective layers.

**Table 1:** Performance of selected SHJ solar cells featuring nanocrystalline silicon based thin films as carrier-selective contacts

Layer/stack	Focused area of investigation	Significant result	Cell parameters	Ref.
nc-SiO_*x*_:H(p)	Reducing optical reflection losses at the front side	Photocurrent density enhancement	*V*_OC_ = 688 mV, *J*_SC_ = 40.4 mA cm^−2^, FF = 72.9%, *η* = 20.3%	23
nc-SiO_*x*_:H(n)	To minimise current losses	Fill factor improvement by reducing resistance losses	*V*_OC_ = 729 mV, *J*_SC_ = 40 mA cm^−2^, FF = 80.0%, *η* = 21.6%	61
nc-Si:H(p)/nc-SiO_*x*_:H(p)	Emitter/TCO contact resistance loss minimization	Fill factor improvement with the better carrier tunnelling	*V*_OC_ = 696 mV, *J*_SC_ = 37.5 mA cm^−2^, FF = 76.8%, *η* = 20.2%	67
	nc-Si:H(p) contact layer			
nc-Si:H(n,p)	Complete device fabrication with the doped nc-Si:H layers	Improvement in optical and electrical performance of a device	*V*_OC_ = 721 mV, *J*_SC_ = 36.9 mA cm^−2^, FF = 79.3%, *η* = 21.1%	15
nc-SiO_*x*_:H(n)	Ultrathin (5 nm) nc-SiO_*x*_:H front emitter	Short deposition time (<100 s)	*V*_OC_ = 731 mV, *J*_SC_ = 38.3 mA cm^−2^, FF = 80.6%, *η* = 22.6%	62
	nc-Si:H seed and contact layers			
nc-Si:H(p)	Plasma pre-treatment of the a-Si:H(i) layer	Improved crystallinity and transparency of the nc-Si:H(p) layers	*V*_OC_ = 734.1 mV, *J*_SC_ = 39.41 mA cm^−2^, FF = 81.07%, *η* = 23.45%	65
	Low-temperature deposition of nc-Si:H(p)	Higher band bending in the c-Si wafer		
nc-SiO_*x*_(n)	Front contact stack	Full-size (244 cm^2^) rear-junction cell	*V*_OC_ = 739 mV, *J*_SC_ = 38.7 mA cm^−2^, FF = 80.7%, *η* = 23.1%	24
	Effect of nc-Si:H seed layer and contact layer	FF exceeding 80% for the front stack having a 5 nm nc-SiO_*x*_:H(n) and 5 nm nc-Si:H(n) contact		
nc-SiO_*x*_(n)	Front surface field (20 nm)	Full-area (244 cm^2^) rear-emitter cell exceeding 25% efficiency	*V*_OC_ = 747 mV, *J*_SC_ = 39.55 mA cm^−2^, FF = 84.98%, *η* = 25.11%	25
	Bilayer intrinsic a-Si:H passivation			

Although the nc-Si:H layers have carrier transport benefits, a few challenges remain associated with incorporating the doped nc-Si:H layers. One of the problems is the doping concentration limitation, mainly when designing a p-type layer with boron dopants, which inhibits crystallites' growth due to structural hindrance from the substitutional doping.^68–70^ The challenge is also the nucleation of the nc-Si:H layer on top of the intrinsic a-Si:H passivation layer without affecting it.^55^ Often, thicker (10–20 nm) doped nc-Si films are required for better transport characteristics of the SHJ cell, which limit its adoption in industrial manufacturing because of a high deposition time. The plasma pre-treatment of the intrinsic a-Si:H passivation layer has been suggested for instantaneous nucleation and enhancing the crystallinity of the very thin nc-Si:H layers.^71,72^ Some of the significant observations with the plasma treatments are discussed in the following section.

### 3.3 Hydrogen/carbon dioxide plasma treatment

The interface pre-treatment improves the passivation quality through surface restructuring as well as fast nucleation of the doped nc-Si layer.^70–74^ The hydrogen present during post-deposition acts as a catalyst (as well as an etchant) for promoting the surface reactions to facilitate crystallisation of the silicon matrix by restructuring the Si–Si bonds, apart from reducing the grain boundary defects. This process involves two steps: initial formation of intermediate Si–H–Si bonds and then reconstruction/relaxation into Si–Si bonds with the bond angles and lengths close to those of the (nano)crystalline silicon.^35^ Guha *et al.*^75^ have reported the threshold hydrogen dilution condition for the transition from the μc-Si:H to the a-Si:H structure. Pham *et al.*^76^ emphasised the role of the undoped nc-Si:H seed layer combined with the post hydrogen treatment in promoting the crystallinity of the front nc-Si:H(n) layer and also demonstrated an improvement in layer conductivity which resulted in the better performance of the final device. A recent investigation showed that the combined process improvement of the hydrogen plasma treatment and the very high frequency (VHF) plasma deposition of the nc-SiO_*x*_:H layer also led to a cell power conversion efficiency of ∼22%.^77^

Mazzarella *et al.* have investigated the CO_2_ plasma pre-treatment of the i-layer prior to the p-type nc-Si:H layer growth and the effect of its thickness on a cell's fill factor and series resistance.^71^ The treatment was shown to be responsible for suppressing the weak Si–Si/Si–H bonds in a layer with the Si–O bonds, which promotes structural order. Vaucher *et al.*^72^ have reported the active nucleation and the associated improvements in the cell's open-circuit voltage due to the CO_2_ plasma pre-treatment. Boccard *et al.*^78^ have explored the nucleation of the μc-Si:H layer on the intrinsic a-Si:H layer with the CO_2_ concentration variation and treatment time. Fioretti *et al.*^65^ have reported a ∼23.45% efficient SHJ cell with a low-temperature (175 °C) deposited p-type nc-Si:H emitter layer combined with the plasma pre-treatment of the underlying a-Si:H(i) layer. It was identified that the low-temperature deposition led to a marked improvement in crystallinity (from 35% to 55%) with the better percolation of crystallites along the growth direction, providing easy transport of photo-generated charge carriers. The role of stacked front n-type layer (comprising seed nc-Si:H, bulk nc-SiO_*x*_:H and contact nc-Si:H layers) for augmenting the SHJ device performance combined with the plasma pre-treatment of the a-Si:H(i) layer has also been investigated, and has highlighted the importance of a non-oxidic seed layer for enhanced nucleation of nc-SiO_*x*_ film.^62^ The work also highlighted a reduced deposition time (<100 s) for the front stack, wherein ultrathin 5 nm nc-SiO_*x*_:H(n) combined with an nc-Si:H(n) contact was shown to give the best cell efficiency of 22.6%, with FF exceeding 80%.^22^ Similarly, the scalability and homogeneity of the ultrathin n-type stack as a front contact has been demonstrated on a large-area (244 cm^2^) SHJ cell, whereby an efficiency of 23.1% has been demonstrated by implementing the stack of 5 nm nc-SiO_*x*_:H(n) and 5 nm nc-Si:H(n) front contact layers.^24^

From the above discussion, it can be seen that research on the doped nc-Si:H layers as carrier-selective contacts in SHJ cells is rapidly evolving, as demonstrated by recent promising cell performances shown in Table 1. Several challenges remain to be solved as routine SHJ cell efficiencies exceeding 25% and *V*_OC_ well above 740 mV are yet to be demonstrated. Adoption of p-type nc-Si:H as front contacts seems to be difficult, as a trade-off has to be made between the final SHJ cell parameters, such as FF and *J*_SC_. Stacking of p-type nc-SiO_*x*_:H layers and ultrathin nc-Si:H(p) contact layers seems to be the better option to improve the p/TCO contact properties whereby a *J*_SC_ value of 40 mA cm^−2^ could be achieved. The rear-emitter SHJ configuration opens wide possibilities by allowing the superior n-type nc-Si:H or nc-SiO_*x*_:H as front contacts that show better characteristics. Doped nc-SiO_*x*_:H layers as the front surface field, especially a stack of nc-Si/nc-SiO_*x*_, are promising because of the impressive *J*_SC_ values, and its tunable properties and excellent SHJ cell performances have been demonstrated on a laboratory scale as well as on industrial silicon wafers. At the same time, the rear-emitter configuration requires wide adoption of thin n-Cz wafers with a high bulk lifetime (>3 ms) for the holes to be collected at the rear. Still, deposition engineering combined with understanding the material and interface properties is needed to fully address some of the issues.

## 4. Conclusions

The growth, material properties, and current status of nc-Si layers for the SHJ solar cell application are reviewed. The literature related to the growth of nc-Si:H in the a-Si:H matrix *via* the surface and growth zone models is emphasised. The surface model is suggested to have a limitation in explaining the thicker films with sufficient crystallinity; thus, the growth zone model has been considered to be suitable for discussing the surface as well as subsurface growth reactions. The overall understanding of the nc-Si:H films' growth is based on (1) surface and growth zone models, and (2) pre-/post-deposition (H_2_/CO_2_) plasma treatment to influence the incubation of crystallinity by promoting subsurface reactions to initiate fast nucleation, and also a change from a disordered to an ordered structure.

We also briefly presented the nc-Si:H layers' advantage as the carrier-selective layers for the SHJ cells to overcome some of the optical and electrical losses in comparison to the conventional a-Si:H layers. The oxygen alloying of the nc-Si:H (emitter) layer is found to be the better option for controlling the parasitic absorption loss with a modified refractive index instead of pure nc-Si:H layers. The H_2_/CO_2_ plasma treatment for the nc-Si:H layers is discussed for promoting charge carrier transport through percolation of crystallites with different process condition variations (plasma frequency and low-temperature deposition). Still, there is much scope in this emerging area for better understanding of the nc-Si:H growth on c-Si with different orientations and plasma treatments, and further related to the carrier transport mechanisms and energy band offsets at the interface based on nc-Si:H layers as the carrier-selective layers in the SHJ device.

## Author contribution

Mansi Sharma: conceptualisation, investigation, writing – original draft preparation, reviewing and editing. Jagannath Panigrahi: visualisation, validation, writing – reviewing and editing. V. K. Komarala: conceptualisation, resources, supervision, writing – reviewing and editing.

## Conflicts of interest

There are no conflicts to declare.

## Supplementary Material

## References

[cit1] Haegel N. M. (2019). *et al.*, Terawatt-scale photovoltaics: Transform global energy. Science.

[cit2] Stuckelberger M., Biron R., Wyrsch N., Haug F. J., Ballif C. (2017). Review: Progress in solar cells from hydrogenated amorphous silicon. Renewable Sustainable Energy Rev..

[cit3] Adachi D., Hernandez J. L., Yamamoto K. (2015). Impact of carrier recombination on fill factor for large area heterojunction crystalline silicon solar cell with 25.1% efficiency. Appl. Phys. Lett..

[cit4] Yoshikawa K., Kawasaki H., Yoshida W., Irie T., Konishi K., Nakano K., Uto T., Adachi D., Kanematsu M., Uzu H., Yamamoto K. (2017). Silicon heterojunction solar cell with interdigitated back contacts for a photoconversion efficiency over 26%. Nat. Energy.

[cit5] Holman Z. C., Descoeudres A., Barraud L., Fernandez F. Z., Seif J. P., De Wolf S., Ballif C. (2012). Current losses at the front of silicon heterojunction solar cells. IEEE J. Photovolt..

[cit6] Holman Z. C., Filipic M., Descoeudres A., De Wolf S., Smole F., Topic M. (2013). *et al.*, Infrared light management in high efficiency silicon heterojunction and rear-passivated cells. J. Appl. Phys..

[cit7] Fujiwara H., Kondo M. (2007). Effects of a-Si:H layer thicknesses on the performance of a-Si:H/c-Si heterojunction solar cells. J. Appl. Phys..

[cit8] Holman Z. C., Descoeudres A., Barraud L., Fernandez F. Z., Seif J. P., De Wolf S., Ballif C. (2013). Record infrared internal quantum efficiency in silicon heterojunction solar cells with dielectric/metal rear reflectors. IEEE J. Photovolt..

[cit9] De Wolf S., Kondo M. (2009). Nature of doped a-Si:H/c-Si interface recombination. J. Appl. Phys..

[cit10] Bivour M., Reichel C., Hermle M., Glunz S. W. (2012). Improving the a-Si:H(p) rear emitter contact of n-type silicon solar cells. Sol. Energy Mater. Sol. Cells.

[cit11] Fujiwara H., Kaneko T., Kondo M. (2007). Application of hydrogenated amorphous silicon oxide layers to c-Si heterojunction solar cells. Appl. Phys. Lett..

[cit12] Mueller T., Wong J., Aberle A. G. (2012). Heterojunction thin film solar cells using amorphous silicon suboxides for interface passivation. Energy Procedia.

[cit13] Ghahfarokhi O. M., von Maydell K., Agert C. (2014). Enhanced passivation at amorphous/crystalline silicon interface and suppressed Schottky barrier by deposition of microcrystalline silicon emitter layer in silicon heterojunction solar cells. Appl. Phys. Lett..

[cit14] Watahiki T. (2015). *et al.*, Rear-emitter Si heterojunction solar cells with over 23% efficiency. Appl. Phys. Express.

[cit15] Nogay G., Seif J. P., Riesen Y., Tomasi A., Jeangros Q., Wyrsch N., Haug F. J., De Wolf S., Ballif C. (2016). Nanocrystalline silicon carrier collectors for silicon heterojunction solar cells and impact on low-temperature device characteristics. IEEE J. Photovolt..

[cit16] Ling Z. P., Ge J., Mueller T., Wong J., Aberle A. G. (2012). Optimisation of p-doped μc-Si:H emitter layers in crystalline-amorphous silicon heterojunction solar cells. Energy Procedia.

[cit17] Shah A. V., Meier J., Vallat-Sauvain E., Wyrsch N., Kroll U., Droz C., Graf U. (2003). Material and solar cell research in microcrystalline silicon. Sol. Energy Mater. Sol. Cells.

[cit18] i Cabarrocas P. R. (2004). New approaches for the production of nano-, micro-, and polycrystalline silicon thin films. Phys. Status Solidi C.

[cit19] Fathi E., Vygranenko Y., Vieira M., Sazonov A. (2011). Boron-doped nanocrystalline silicon thin films for solar cells. Appl. Surf. Sci..

[cit20] Lambertz A., Smirnov V., Merdzhanova T., Ding K., Haas S., Jost G., Schropp R. E. I., Finger F., Rau U. (2013). Microcrystalline silicon-oxygen alloys for application in silicon solar cells and modules. Sol. Energy Mater. Sol. Cells.

[cit21] Ding K., Aeberhard U., Finger F., Rau U. (2012). Silicon heterojunction solar cell with amorphous silicon oxide buffer and microcrystalline silicon oxide contact layers. Phys. Status Solidi RRL.

[cit22] Mazzarella L., Kirner S., Gabriel O., Korte L., Stannowski B., Rech B., Schlatmann R. (2015). Nanocrystalline Silicon Oxide Emitters for Silicon Hetero Junction Solar Cells. Energy Procedia.

[cit23] Mazzarella L., Kirner S., Stannowski B., Korte L., Rech B., Schlatmann R. (2015). p-type microcrystalline silicon oxide emitter for silicon heterojunction solar cells allowing current densities above 40 mA/cm^2^. Appl. Phys. Lett..

[cit24] Qiu D., Duan W., Lambertz A., Bittkau K., Steuter P., Liu Y., Gad A., Pomaska M., Rau U., Ding K. (2020). Front contact optimisation for rear-junction SHJ solar cells with ultra-thin n-type nanocrystalline silicon oxide. Sol. Energy Mater. Sol. Cells.

[cit25] Ru X., Qu M., Wang J., Ruan T., Yang M., Peng F., Long W., Zheng K., Yan H., Xu X. (2020). 25.11% efficiency silicon heterojunction solar cell with low deposition rate intrinsic amorphous silicon buffer layers. Sol. Energy Mater. Sol. Cells.

[cit26] Juneja S., Sudhakar S., Gope J., Lodhi K., Sharma M., Kumar S. (2015). Highly condcutive boron doped micro/nanocrystalline silicon thin films deposited by VHF-PECVD for solar cell applications. J. Alloys Compd..

[cit27] De Yoreo J. J., Vekilov P. G. (2003). Principles of Crystal Nucleation and Growth. Rev. Mineral. Geochem..

[cit28] Juneja S., Sudhakar S., Srivastava A. K., Kumar S. (2016). Morphology and micro-structural studies of distinct silicon thin films dposited using very high frequency plasma enhanced chemical vapor deposition process. Thin Solid Films.

[cit29] Gope J., Kumar S., Parashar A., Dixit P. N., Rauthan C. M. S., Panwar O. S., Patel D. N., Agarwal S. C. (2009). Amorphous and nanocrystalline silicon made by varying deposition pressure in PECVD process. J. Non-Cryst. Solids.

[cit30] Matsuda A. (2004). Thin-film silicon - Growth process and solar cell application. Jpn. J. Appl. Phys..

[cit31] Guo L., Kondo M., Fukawa M., Saitoh K., Matsuda A. (1998). High rate deposition of microcrystalline silicon using conventional plasma-enhanced chemical vapor deposition. Jpn. J. Appl. Phys..

[cit32] Veneri P. D., Mercaldo L. V., Minarini C., Privato C. (2004). VHF PECVD microcrystalline silicon: From material to solar cells. Thin Solid Films.

[cit33] Rath J. K., Franken R. H. J., Gordijn A., Schropp R. E. I., Goedheer W. J. (2004). Growth mechanism of microcrystalline silicon at high pressure conditions. J. Non-Cryst. Solids.

[cit34] Layadi N., i Cabarrocas P. R., Drévillon B., Solomon I. (1995). Real-time spectroscopic ellipsometry study of the growth of amorphous and microcrystalline silicon thin films prepared by alternating silicon deposition and hydrogen plasma treatment. Phys. Rev. B.

[cit35] Sriraman S., Agarwal S., Aydil E. S., Maroudas D. (2002). Mechanism of hydrogen-induced crystallisation of amorphous silicon. Nature.

[cit36] Mahan A. H., Ahrenkiel S. P., Schropp R. E. I., Li H., Ginley D. S. (2008). A comparison of grain nucleation and grain growth during crystallisation of HWCVD and PECVD a-Si:H films. Thin Solid Films.

[cit37] Kahn H., He A. Q., Heuer A. H. (2002). Homogeneous nucleation during crystallisation of amorphous silicon produced by low-pressure chemical vapour deposition. Philos. Mag. A.

[cit38] Shah A. (2000). Intrinsic microcrystalline silicon (μc-Si:H) deposited by VHF-GD (very high frequency-glow discharge): A new material for photovoltaics and optoelectronics. Mater. Sci. Eng., B.

[cit39] i Cabarrocas P. R., Layadi N., Heitz T., Drévillon B., Solomon I. (1995). Substrate selectivity in the formation of microcrystalline silicon: Mechanisms and technological consequences. Appl. Phys. Lett..

[cit40] Tsai C. C., Anderson G. B., Thompson R., Wacker B. (1989). Control of silicon network structure in plasma deposition. J. Non-Cryst. Solids.

[cit41] Solomon I., Drévillon B., Shirai H., Layadi N. (1993). Plasma deposition of microcrystalline silicon: the selective etching model. J. Non-Cryst. Solids.

[cit42] Kirner S., Gabriel O., Stannowski B., Rech B., Schlatmann R. (2013). The growth of microcrystalline silicon oxide thin films studied by *in situ* plasma diagnostics. Appl. Phys. Lett..

[cit43] Shibata N., Fukuda K., Ohtoshi H., Hanna J. I., Oda S., Shimizu I. (1987). Preparation of polycrystalline silicon by hydrogen-radical-enhanced chemical vapor deposition. Jpn. J. Appl. Phys..

[cit44] Yang Y. H., Katiyar M., Feng G. F., Maley N., Abelson J. R. (1994). Subsurface hydrogenated amorphous silicon to μc-hydrogenated silicon transformation during magnetron sputter deposition determined by spectroscopic ellipsometry. Appl. Phys. Lett..

[cit45] Descoeudres A., Barraud L., De Wolf S., Strahm B., Lachenal D., Guérin C., Holman Z. C., Zicarelli F., Demaurex B., Seif J., Holovsky J. (2011). Improved amorphous/crystalline silicon interface passivation by hydrogen plasma treatment. Appl. Phys. Lett..

[cit46] Watanabe Y., Shiratani M., Kubo Y., Ogawa I., Ogi S. (1988). Effects of low-frequency modulation on rf discharge chemical vapor deposition. Appl. Phys. Lett..

[cit47] Hishida M., Sekimoto T., Matsumoto M., Terakawa A. (2016). Intermittent very high frequency plasma deposition on microcrystalline silicon solar cells enabling high conversion efficiency. Energies.

[cit48] Sharma M., Chaudhary D., Sudhakar S., Jadkar S., Kumar S. (2018). Investigation on sub nano-crystalline silicon thin films grown using pulsed PECVD process. Mater. Sci. Semicond. Process..

[cit49] Sharma M., Chaudhary D., Sudhakar S., Kumar S. (2020). Intrinsic Sub-Nanocrystalline Silicon Thin Films: Active Layer for Solar Cells. Silicon.

[cit50] De Wolf S., Descoeudres A., Holman Z. C., Ballif C. (2012). High-efficiency silicon heterojunction solar cells: A review. Green.

[cit51] Fujiwara H., Kondo M. (2007). Impact of epitaxial growth at the heterointerface of a-si: H∕c-si solar cells. Appl. Phys. Lett..

[cit52] De Wolf S., Kondo M. (2007). Abruptness of a-Si:H/c-Si interface revealed by carrier lifetime measurements. Appl. Phys. Lett..

[cit53] Bivour M., Schröer S., Hermle M., Glunz S. W. (2014). Silicon heterojunction rear-emitter solar cells: Less restrictions on the optoelectrical properties of front side TCOs. Sol. Energy Mater. Sol. Cells.

[cit54] Melskens J., Van De Loo B. W. H., Macco B., Black L. E., Smit S., Kessels W. M. M. (2018). Passivating Contacts for Crystalline Silicon Solar Cells: From Concepts and Materials to Prospects. IEEE J. Photovolt..

[cit55] Seif J. P., Descoeudres A., Nogay G., Hänni S., De Nicolas S. M., Holm N., Geissbühler J., Hessler-Wyser A., Duchamp M., Dunin-Borkowski R. E., Ledinsky M., De Wold S., Ballif C. (2016). Strategies for doped nanocrystalline silicon integration in silicon heterojunction solar cells. IEEE J. Photovolt..

[cit56] Street R. (1985). Localised states in doped amorphous silicon. J. Non-Cryst. Solids.

[cit57] Kumar S., Dixit P. N., Rauthan C. M. S., Parashar A., Gope J. (2008). Effect of power on the growth of nanocrystalline silicon films. J. Phys.: Condens. Matter.

[cit58] Das D., Bhattacharya K. (2016). Characterisation of the Si:H network during transformation from amorphous to micro- and nanocrystalline structures. J. Appl. Phys..

[cit59] Parashar A., Kumar S., Gope J., Rauthan C. M. S., Dixit P. N., Hashmi S. A. (2010). Influence of argon dilution on growth and properties of hydrogenated nanocrystalline silicon films. Sol. Energy Mater. Sol. Cells.

[cit60] Vasiliev I., Öǧüt S., Chelikowsky J. R. (2001). Ab initio absorption spectra and optical gaps in nanocrystalline silicon. Phys. Rev. Lett..

[cit61] Mazzarella L., Morales-Vilches A. B., Hendrichs M., Kirner S., Korte L., Korte L., Schlatmann R., Stannowski B. (2018). Nanocrystalline n-type silicon oxide front contacts for silicon heterojunction solar cells: photocurrent enhancement on planar and textured substrates. IEEE J. Photovolt..

[cit62] Mazzarella L., Morales-Vilches A. B., Korte L., Schlatmann R., Stannowski B. (2018). Ultra-thin nanocrystalline n-type silicon oxide front contact layers for rear-emitter silicon heterojunction solar cells. Sol. Energy Mater. Sol. Cells.

[cit63] Shimakawa K. (2000). Percolation-controlled electronic properties in microcrystalline silicon: effective medium approach. J. Non-Cryst. Solids.

[cit64] Richter A., Smirnov V., Lambertz A., Nomoto K., Welter K., Ding K. (2018). Versatility of doped nanocrystalline silicon oxide for applications in silicon thin-film and heterojunction solar cells. Sol. Energy Mater. Sol. Cells.

[cit65] Fioretti A. N., Boccard M., Monnard R., Ballif C. (2019). Low-Temperature p-Type Microcrystalline Silicon as Carrier Selective Contact for Silicon Heterojunction Solar Cells. IEEE J. Photovolt..

[cit66] Matsui T., Kondo M., Matsuda A. (2004). Doping properties of boron-doped microcrystalline silicon from B_2_H_6_ and BF_3_: material properties and solar cell performance. J. Non-Cryst. Solids.

[cit67] Kirner S., Mazzarella L., Korte L., Stannowski B., Rech B., Schlatmann R. (2015). Silicon Heterojunction Solar Cells with Nanocrystalline Silicon Oxide Emitter: Insights into Charge Carrier Transport. IEEE J. Photovolt..

[cit68] Hao X. J., Cho E. C., Flynn C., Shen Y. S., Conibeer G., Green M. A. (2008). Effects of boron doping on the structural and optical properties of silicon nanocrystals in a silicon dioxide matrix. Nanotechnology.

[cit69] Saleh R., Nickel N. H. (2007). The influence of boron concentrations on structural properties in disorder silicon films. Appl. Surf. Sci..

[cit70] Koh J., Fujiwara H., Koval R. J., Wronski C. R., Collins R. W. (1999). Real time spectroscopic ellipsometry studies of the nucleation and growth of p-type microcrystalline silicon films on amorphous silicon using B_2_H_6_, B(CH_3_)_3_ and BF_3_ dopant source gases. J. Appl. Phys..

[cit71] Mazzarella L., Kirner S., Gabriel O., Schmidt S. S., Korte L., Stannowski B., Rech B., Schlatmann R. (2017). Nanocrystalline silicon emitter optimisation for Si-HJ solar cells: Substrate selectivity and CO_2_ plasma treatment effect. Phys. Status Solidi A.

[cit72] Vaucher N. P., Rech B., Fischer D., Dubail S., Goetz M., Keppner H., Wyrsch N., Beneking C., Hadjadj O., Shklover V., Shah A. (1997). Controlled nucleation of thin microcrystalline layers for the recombination junction in a-Si stacked cells. Sol. Energy Mater. Sol. Cells.

[cit73] Vetterl O., Hülsbeck M., Wolff J., Carius R., Finger F. (2003). Preparation of microcrystalline silicon seed-layers with defined structural properties. Thin Solid Films.

[cit74] Zhou J. H., Ikuta K., Yasuda T., Umeda T., Yamasaki S., Tanaka K. (1997). Growth of amorphous-layer-free microcrystalline silicon on insulating glass substrates by plasma-enhanced chemical vapor deposition. Appl. Phys. Lett..

[cit75] Guha S., Yang J., Banerjee A., Yan B., Lord K. (2003). High quality amorphous silicon materials and cells grown with hydrogen dilution. Sol. Energy Mater. Sol. Cells.

[cit76] Pham D. P., Kim S., Lee S., Le A. H. T., Cho E. C., Park J., Yi J. (2019). a crystalline seed layer for mirocrystalline silicon oxide window layer in rear emitter silicon heterojunction cells. Infrared Phys. Technol..

[cit77] Zhao Y., Mazzarella L., Procel P., Han C., Yang G., Weeber A., Zeman M., Isabella O. (2020). Doped hydrogenated nanocrystalline silicon oxide layers for high-efficiency c-Si heterojunction solar cells. Prog. Photovoltaics.

[cit78] Boccard M., Monnard R., Antognini L., Ballif C. (2018). Silicon oxide treatment to promote crystallinity of p-type microcrystalline layers for silicon heterojunction solar cells. AIP Conf. Proc..

